# Friend or foe? Biological and ecological traits of the European ash dieback pathogen *Hymenoscyphus fraxineus* in its native environment

**DOI:** 10.1038/srep21895

**Published:** 2016-02-22

**Authors:** Michelle Cleary, Diem Nguyen, Diana Marčiulynienė, Anna Berlin, Rimvys Vasaitis, Jan Stenlid

**Affiliations:** 1 Southern Swedish Forest Research Centre, Swedish University of Agricultural Sciences, Alnarp, Sweden, Box 49, Sundsvägen 3, SE-23053 Alnarp, Sweden; 2 Department of Forest Mycology and Plant Pathology, Swedish University of Agricultural Sciences, Box 7026, Almas allé 5, SE-75007 Uppsala, Sweden; 3 Institute of Forestry, Lithuanian Research Centre for Agriculture and Forestry, Liepų str. 1, LT-53101 Girionys, Kaunas distr., Lithuania

## Abstract

*Hymenoscyphus fraxineus*, an introduced ascomycete fungus and primary causal agent of European ash dieback, was investigated on *Fraxinus mandshurica* trees in its native range in Primorye region of Far East Russia. This evidence is the first report of *H. fraxineus* on healthy, asymptomatic *F. mandshurica* trees. High-throughput sequencing revealed 49 distinct fungal taxa associated with leaves of *F. mandshurica*, 12 of which were identified to species level. Phyllosphere fungal assemblages were similar among sites despite being largely geographically distant. Many organisms comprising the foliar fungal community on *F. mandshurica* in Far East Russia have similarity to those reported inhabiting *F. excelsior* in Europe based on previous studies. However, *Mycosphaerella* sp., the most dominant species in this study and detected in nearly all samples, was associated only with *F. mandshurica*. Genetic diversity of *H. fraxineus* was significantly higher in the Far East Russian population than in Europe. In contrast to its aggressive behaviour on *Fraxinus excelsior* in Europe, *H. fraxineus* appears to be a benign associate of indigenous *F. mandshurica* that initially induces quiescent and asymptomatic infections in healthy trees prior to active host colonization normally associated with modification of host tissue during senescence.

A new alien invasive disease affecting common ash (*Fraxinus excelsior*) trees has resulted in severe dieback and mortality throughout much of the natural distribution range of ash in northern and central Europe. The causal agent of disease was first described as *Chalara fraxinea*, characterized by brown phialides producing conidia in slimy spore droplets, and occasionally in chains^1^. Its teleomorph was mistakenly recognized as *Hymenoscyphus albidus*, a discomycete native to Europe that produces numerous white-stalked apothecia on fallen rachises, and which had never been reported to be pathogenic^2^. Differences in the genomic DNA markers internal transcribed spacer (ITS), elongation factor-1-α (EF1-α), and calmodulin (CAL) gene and inter simple sequence repeat-polymerase chain reaction (ISSR-PCR) fingerprinting of specimens collected from diseased and healthy ash stands clearly delimited two species that appeared morphologically indistinct^3^. The separate cryptic telemorph species associated with the anamorph *C. fraxinea* was subsequently named *Hymenoscyphus pseudoalbidus*^3^. Subsequent work has demonstrated subtle microscopic differences between the two *Hymenoscyphus* species on the basis of presence or absence of ascus croziers^4^. Recently, a suggested renaming of the fungus to *H. fraxineus* Baral, Queloz, Hosoya was proposed^5^.

Apothecia of *H. fraxineus* are produced during the summer mainly on blackened pseudosclerotial rachises from the previous year in the leaf litter^6^,^7^,^8^. Occasionally apothecia have been noted also on small ligneous stems^2^,^7^. Following spore dispersal during several weeks in the summer, germ tube formation and development of appressorium facilitate penetration of the ash leaf cuticle^8^ and necrosis expands proximally preferentially along leaf veins to rachises and subsequently twigs and branches leading to a wide range of symptoms including wilting of shoots, bark cankers, wood discoloration and dieback of twigs, branches and the crown^9^,^10^.

Population studies of *H. fraxineus* in Europe show high genotypic diversity^11^,^12^ suggesting an outbreeding mating system and long-range dispersal via ascospores, and reported low allelic richness and low differentiation among European populations of *H. fraxineus*^13^,^14^,^15^. Collectively the different studies conducted throughout Europe suggest no population structure, and that the pathogen must have gone through a strong genetic bottleneck in the European populations^12^,^13^,^14^,^15^, with the exception of some differentiation between the Polish highland and lowland populations^11^. *H. fraxineus* in Europe is conspecific with Japanese specimens^16^, previously known under the name *Lambertella albida* (≡ *H. albidus*) collected from decaying rachises of *F. mandshurica* var. *japonica*. The Asian *H. fraxineus* population showed higher genetic diversity and was genetically differentiated from the European population^17^ suggesting the origin of the pathogen introduced to Europe is likely East Asia.

In Russia, there is large geographic separation between natural distribution ranges of *F. excelsior* in the west and Manchurian ash (*F. mandshurica*) in the east. In Russia’s Kaliningrad district, which is approximately in the far eastern range of *F. excelsior*’s natural distribution, *H. fraxineus* is well established having spread from diseased areas in nearby Baltic countries, and is causing decline and mortality of trees^18^. *Fraxinus sogdiana*, a species of middle Asia, was shown to be susceptible to *H. fraxineus* in Krussmann’s ash belt in Central Asia^19^. In Far East Russia, *F. mandshurica* grows in natural mixed forests of Primorye region which represents the north-eastern and part of the central area of the disjunctive natural range of the species in the East^20^. The ecology of *H. fraxineus* on *F. mandshurica* and other *Fraxinus* species within its native environment in East Asia however still remains unclear. Contrasting reports of disease expression exist between amenity plantings of *F. mandshurica* within the current zone of infestation in Europe and that within its native range in the East. First, there have been no reports of leaf symptoms on *F. mandshurica* in its native range of Asia that would suggest *H. fraxineus* is a pathogen on *F. mandshurica*. Yet following artificial stem-wound inoculations on seedlings, *H. fraxineus* was shown to be pathogenic on *F. mandshurica*^21^. In Estonia, typical symptoms of ash dieback were reported on 40-year-old *F. mandschurica* planted in a park and *H. fraxineus* was consistently isolated from shoots of trees displaying these symptoms^22^, though it was noted that among the non-native *Fraxinus* species, *F. mandshurica* and *F. americana* were the least affected with only minor shoot and twig dieback and bark necrosis. The above reports^21^,^22^ contrast with surveys of a number of Asian *Fraxinus* species, including *F. mandshurica*, planted alongside severely damaged *F. excelsior* in southern Swedish arboreta (Alnarp, Göteborg) which showed no wilting, bark necrosis or dieback of twigs and branches (Fig. 1) (M. Cleary, unpublished data). Similar observations of disease-free Asian *Fraxinus* have been reported from arboreta in Denmark^23^. *H. fraxineus* is found on shed pseudosclerotial rachises of *F. mandshurica*, and its suggested role is as a decomposer of ash leaves in the leaf litter^16^. Since pathogenicity to indigenous *Fraxinus* species in the East has so far not been reported under natural conditions, one may speculate that *H. fraxineus* in its native environment has a primary role as a saprotroph of senesced leaf tissue, but possesses a prolonged period of endophytic growth prior to saprobic colonization of host tissue normally associated with senescence.

Phyllosphere fungal species include both epiphytes (organisms living on the host surface) and endophytes. The vast majority of plants in natural ecosystems are colonized by endophytes which generally reside asymptomatically in apoplastic spaces and/or within the living cells of plants for all or at least a part of their life cycle by means of quiescent infections^24^. Phyllosphere fungi have diverse roles influencing plant fitness either negatively as pathogens^25^, or conferring benefits on their hosts by increasing plant’s tolerance to stress^26^, reducing herbivory through the production of toxic alkaloids^27^, and via antagonistic effects that reduce infection of plant tissues by pathogens^28^. Such roles are of course dependent on any large number of variables including the affected host plant species and environmental parameters that condition the host for infection. Earlier studies have revealed a large diversity of phyllosphere-inhabiting microorganisms of both healthy and diseased *F. excelsior*^9^,^10^,^29^,^30^,^31^,^32^ and profile a range of fungal assemblages for the species. However, little is known of the fungal community associated with *F. mandshurica,* whether large qualitative differences exist between *F. excelsior* in Europe and *F. mandshurica* in its native environment in the East, and whether this could, in part, explain differences in the behaviour of *H. fraxineus* on either *Fraxinus* species.

The aims of the current study were i) to investigate whether *H. fraxineus* is present on healthy (i.e. asymptomatic) leaves of *F. mandshurica* trees located in Far East Russia, ii) to describe the diversity and spatial variability of the associated fungal community of *F. mandshurica* trees, and iii) determine the specific structure of genetic diversity of *H. fraxineus* between populations in Far East Russia and Europe.

## Results

### Fungal community of F. mandshurica and detection of H. fraxineus in asymptomatic leaves

Investigation of fungal communities in asymptomatic *F. mandshurica* leaflets and rachises revealed the presence of 49 distinct fungal taxa, 12 of which were identified to species level based on sequence similarities with GenBank entries. 96% of the sequence reads exist in 20 of the most frequently occurring or dominant OTUs (distinct taxa) that belonged to six orders of Ascomycetes: *Capinodiales*, *Pleosporales*, *Diaporthales*, *Helotiales*, *Hypocreales*, *Dothideales* and an unknown Ascomycete; and to one order of Basidiomycetes, *Tremellales* (Table 1). Of these 20 most frequently occurring OTUs (see also Supplementary Information), seven were identified to the species level and 11 were identified to the genus level. Members of *Capnodiales* and *Pleosporales* dominated the community in both species richness (five species from each order) representing 79% and 12% of OTU relative abundance, respectively. *Mycosphaerella* sp. was the most common species in *F. mandshurica*, and was detected in 99% (n = 75) of samples examined, while *Cladosporium* sp., *Phomopsis* sp., and *Phoma* sp. were found in 84%, 80%, and 81% of samples, respectively. *Hymenoscyphus fraxineus* (Genbank accession no. KU234397) was detected by this method from 33% of the total number of samples examined in all three sites; individual site incidence ranged between 14 and 55% (Table 1).

**Figure 2:**
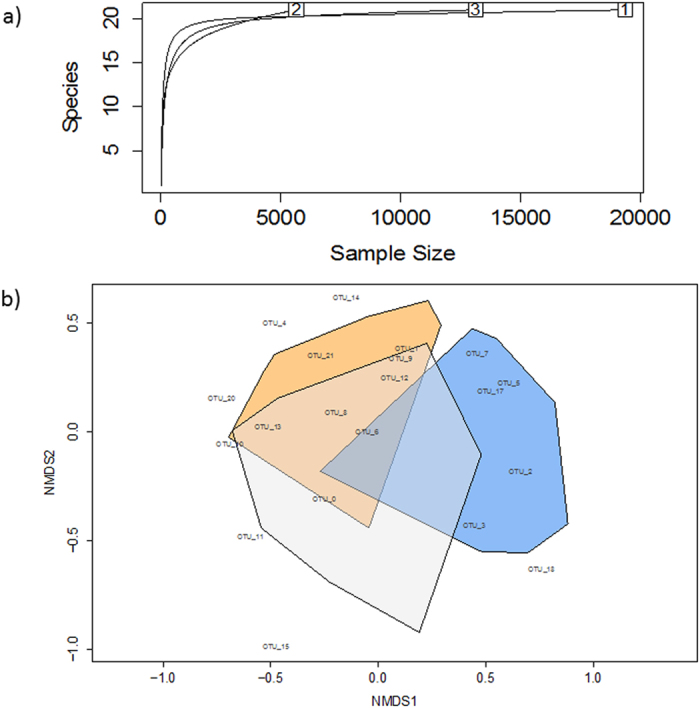
(**a**) Species rarefaction curve and (**b**) nonmetric multidimensional scaling (NMDS) ordination of the top 20 fungal OTUs on *F. mandshurica* at three locations. In (**a**), each boxed number refers to the location of each tree. In (**b**), each convex hull connects the vertices of the points made by each sample from each of the three locations. Orange, blue and grey colors represent individual sites/trees 1, 2, and 3, respectively. The first two dimensions are presented. The final stress value for the NMDS ordination was 0.15.

The nearly overlapping rarefactions curves and overlapping convex hulls in the NMDS indicated that the fungal community was similar at each site (Fig. 2). The Shannon-Wiener Diversity indices of the foliar fungal communities were 2.92, 2.72 and 2.83 for sites 1, 2 and 3, respectively.

To look for similarities in *Fraxinus* fungal communities between Far East Russia and Europe, we compared identified fungal taxa on *F. mandshurica* to recently published literature^9^,^10^,^29^,^30^,^31^,^32^ which report associated fungal taxa to diseased and healthy *F. excelsior* trees. Of the 20 most frequent fungal OTUs in Far East Russia, 12 were similar to species detected on *F. excelsior* in Europe (Table 1). Uniquely associated to *F. mandshurica* were *Coniozyma leucospermi*, *Hannaella coprosmaensis* and species of *Mycosphaerella*, *Dioszegia*, *Paraphoma*, and an unknown *Helotiales*.

### Population genetics of H. fraxineus in Far East Russia versus Europe

**Figure 3:**
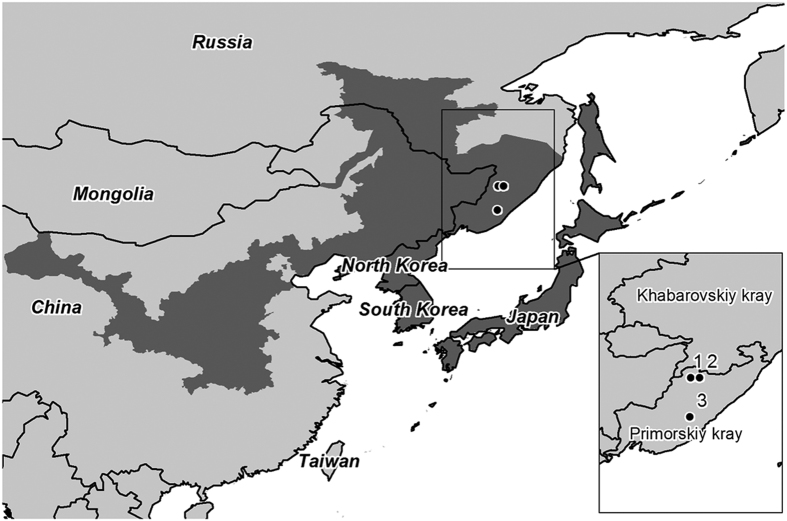
Map showing the approximate natural distribution range of *Fraxinus mandshurica* in East Asia (shaded area) and the three study sites (closed circles) in Primorye region of Far East Russia. Source data for mapping distribution of *F. mandshurica*:^18^,^59^,^60^. Software: ArcGIS version 10.3 (www.esri.com).

The European population was represented from 25 isolates, 15 originating from Sweden and 10 from Lithuania, and the Asian population was represented by 10 samples in Far East Russia, 5 from each of two locations (sites 1 and 3, see Fig. 3). No clones were found among the MLGs in either of two populations. The allelic diversity was larger in Asia than in the European population; the total number of alleles found was 75, of which only 8 were shared; 28 alleles were found in the European population and 57 in the Asian population (Table 2). The numbers of missing alleles were low in the European population; only two loci (Chafra03 and mHp_108810) each had one missing value. The numbers were greater in the Asian population, where half of the samples failed to amplify or showed ambiguous results for loci SSR211 and mHp_108810, and between 2 and 4 of the 10 samples did not amplify for loci SSR38, Chafra04, mHp_080497, mHp_080495 and mHp_079915.

**Figure 4:**
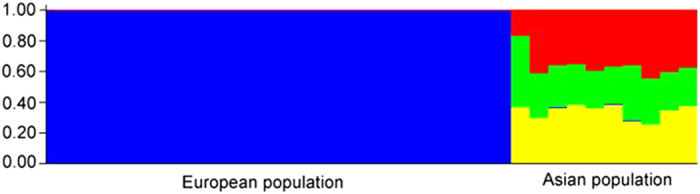
Population structure in Bayesian clustering program Structure (K = 4) for European (i.e. Swedish and Lithuanian) population versus Asian (i.e. Far East Russian) population. The three populations from Asia were not separated among the three different sites.

**Figure 5:**
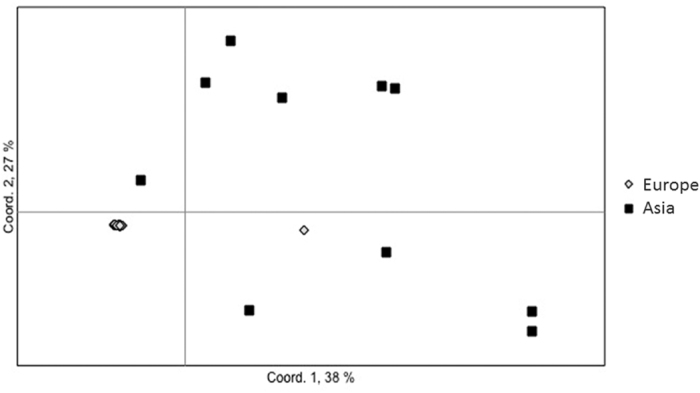
PCoA showing population differences between European and Asian population.

The AMOVA confirmed large differentiation between the two populations, 46% of the molecular variance was found between the European and Asian populations, whereas the remaining 54% was found within the populations (p = 0.0001). The Bayesian clustering analysis of *H. fraxineus* in Structure was based on all 15 loci. The number of populations (K) showing the highest likelihood was 4 (Fig. 4) and it shows a clear division between the European and Asian populations. To further investigate the population differentiation, a PCoA was produced (Fig. 5). The first axis explained 38% and the second axis 27% of the variation between samples. All European samples cluster close together in one group, except for one sample. The only difference with that particular sample is that it has a missing value for locus mHp_108810, which is probably why it is separated from the other European samples. The Asian samples show much larger variation, a few cluster with the European samples and the others show a large genetic variation.

## Discussion

In this study, we used high-throughput DNA sequencing to decipher fungal assemblages inhabiting healthy, asymptomatic *F. mandshurica* leaves. Our results show that individual trees have a highly diverse assemblage (up to 49 distinct fungal taxa detected), though the majority (96%) of OTUs were represented by only 20 taxa, mostly dominated by ascomycetes. Many of the OTUs best matched annotated Gen-Bank accessions of phyllosphere fungi previously detected on plant species other than *Fraxinus*, though not necessarily from the same geographic region as this study. Rarefaction curves and NMDS analysis revealed little intra-host variability in the phyllosphere fungal assemblages, with several overlapping OTUs, despite the large (up to 250 km) geographic separation between sites.

Recent studies describing associated fungi to the foliage and stems of both healthy and diseased *F. excelsior* in Europe^9^,^10^,^29^,^30^,^31^,^32^ were useful in comparing fungal profiles to that of *F. mandshurica* in the East. In many cases similar fungal taxa were detected (Table 1) though few identified at the species level in this study. The most similar taxa included *Cladosporium* sp., *Phomopsis* sp., *Phoma* sp., *Alternaria alternata*, *Cryptococcus foliicola*, *Diaporthe nobilis*, *Periconia byssoides*, *Ramularia* sp. and *Fusarium* sp., which suggests these are more generalist fungal species with a cosmopolitan distribution on a variety of host species.

The ascomycete *H. fraxineus* has been previously documented in Japan^4^,^16^,^33^, north-eastern China^34^, and Korea^35^. Previously *H. fraxineus* has been reported from Primorsky region, Far East Russia on decaying rachises *F. mandshurica*^4^, however this is the first report of its occurrence most notably on healthy, asymptomatic *F. mandshurica* within its natural distribution range in the East. *H. fraxineus* was detected on asymptomatic leaves of *F. mandshurica* at all three sites. Site 3 (Fig. 3) which had a markedly higher frequency of detection on leaves (55%) was also more geographically distant to the other sites. Reasons for the higher level of detection here are not clear, though we cannot exclude the possibility of variable microclimatic conditions among sites that may have influenced both the timing of *H. fraxineus* fruiting on previous year’s rachises, the stage of development of localized infections in host tissue at the time leaves were sampled, or both. Compared to other fungal taxa, *H. fraxineus* had much lower number of sequence reads that may be attributable to several factors including 1) possible DNA degradation during the storage and processing of samples, 2) the timing in which samples were collected (e.g. samples collected late in the season or prior to leaf senescence/leaf shed may exhibit a higher detection frequency than that found in the tested material), 3) endophytic colonization of *H. fraxineus* being restricted to relatively small colonies on the sampled leaves, and 4) antagonistic effects of other more dominant fungi (e.g. *Mycosphaerella* spp., see below) that may actively limit further growth and establishment of *H. fraxineus*.

The most dominant OTU in our study could not be identified to the species level, though the closest GenBank record belonged to *Mycosphaerella* sp. (Table 1). *Mycosphaerella* species are among the largest genera of plant pathogenic fungi that include more than 3000 species and several thousand anamorphs that lack known teleomorph connections^36^. Species of *Mycosphaerella* vary in their ecological role on a wide range of host plants, e.g. as plant pathogens, saprobes, and/or endophytes, though in this study, *Mycosphaerella* sp. was found to be associated with seemingly healthy leaves. Another fungal taxa associated to *F. mandshurica* was *Coniozyma leucospermi*. Ascomycetous fungi with *Coniothyrium*-like anamorphs are common colonisers of wood and leaves of broadleaved plants. They have also been recognized as biological control agents against plant pathogens^37^, and producers of secondary metabolites that may function to inhibit activity related to human diseases^38^. The biological relevance of both of these dominant species to the overall fitness of *F. mandshurica*, its influence on the community composition structure, and in particular its interactions with *H. fraxineus*, warrants further investigation.

The habitat of *H. fraxineus* (syn. *Lambertella albida* s. Hosoya *et al.*) has been described as a saprophyte decaying leaves of *F. mandshurica var*. *japonica*^16^,^33^. This lifestyle behaviour is similar to that of its sister relative *H. albidus* – a well-known decomposer of *F. excelsior* leaves with widespread distribution throughout Europe, but now with rather limited occurrence following the introduction of *H. fraxineus*. In a previous study that characterized the temporal dynamics of the foliar endophytic community of *F. excelsior* throughout the growing season (May–October, during 2008) using isolation and ITS sequencing, *H. albidus* was never detected^30^. This might suggest that *H. albidus* in fact does not establish endophytic infections in leaves of *F. excelsior* during its period of active growth in the same manner that *H. fraxineus* does on *F. mandshurica* within its native range in the East, or that the fungus has been outcompeted by other fungi such that it was not found.

As we have shown in this study, the presence of *H. fraxineus* in asymptomatic leaves of *F. mandshurica* suggests the fungus to possess a biphasic lifestyle, switching from endophytic to saprotrophic life stages on its native host. During its endophytic stage, *H. fraxineus* probably establishes inconspicuous infections on *F. mandshurica* leaves that are highly localized and, at least for some period, in a state of quiescence. In this system, the interactions with *F. mandshurica* may be considered both balanced and antagonistic to explain the apparent symptomless colonization of leaves, in effect resulting in a tolerance of the host to the fungus. Yet it is important to recognize that even balanced antagonistic interactions may be plastic and have the potential for variability and evolutionary development either in the direction of more highly specialized mutualism or parasitism and exploitation^39^. Furthermore, endophytism does not exclude different life history strategies such as the ability to grow saprophytically on dead or senescing tissues following a period of endophytic growth. As an early colonizer with a prolonged endophytic stage, *H. fraxineus* effectively establishes an ‘advantage of position’ that follows with rapid colonization of tissues when suitable conditions are met (e.g. onset of senescence)^40^, and which gives advantage over competing fungi. Innocuous endophytes may also exist as quiescent pathogens^41^, having an unpredictable period of fungal latency, and only produce symptoms and cause disease when the host is subjected to physiological stress^42^; a compatible scenario for trees planted outside their natural distribution range.

Understanding the continuum of endophyte to parasitic lifestyle and the mechanisms involved in determining what makes a particular organism pathogenic on one host and not on another, is undeniably complex. Multiple studies have reported shifts in lifestyles from endophytic to pathogenic stages, and back^43^,^44^,^45^. Frequently, endophytes are sister species to virulent pathogens on the same, or closely related, host species^46^,^47^. Changed abiotic and biotic conditions or single mutations can cause common symptomless endophytes to switch to active growth and saprobic exploitation of the substrate^44^,^48^. A novel host environment may provide little natural resistance to an organism that is flexible in occupying new, yet similar ecological territory – the concept of niche opportunity. Subsequent to this new adaptation, lifestyle changes coincident with the inherent resistance of a new host onto which it successfully ‘jumped’, occurs. Such extreme unprecedented cases, although rare, can result in widespread devastation. A classic example is the Chestnut blight caused by *Cryophonectria parasitica*, a benign associate of Japanese Chestnut (*Castanea crenata*) that was introduced to eastern North America in the early 1900’s. In the 50 years that followed its introduction, North American chestnuts (*Castanea dentata*), which dominated forest ecosystems in eastern North America, were near eliminated.

A more complex issue of concern is the increasing incidence of new invasive pathogens worldwide, our lack of understanding of associated organisms of plants moving through the international plant trade (including lesser known endophytes, and many unknowns), and our ability to predict whether beneficial organisms will have the same mutualistic relationship in a novel niche environment. This is especially relevant for plant quarantines where innocuous phases would not surprisingly be overlooked. Endophytes and pathogens may possess many of the same virulence factors (effectors) necessary to infect and colonize the host to break down leaf components and obtain nutrients to survive as saprotrophs, but these factors are only active in certain life stages. In the case of *H. fraxineus*, it would have been near to impossible to predict that a benign associate of *F. mandshurica*, which before recently was not well characterized, would cause the widespread devastation that engulfs the European population of *F. excelsior* today. Indeed, many forest pathogens now considered invasive in Europe exist in a similar balanced system as having benign associations in their native origin.

In this study we also compared the genetic diversity of the *H. fraxineus* population in Far East Russia to the newly established population of *H. fraxineus* in Europe. Most of the microsatellite markers used in the study were developed based on European populations, and proved to be useful also for the Asian samples. Our results confirm those earlier reported^17^ comparing European and Japanese populations: the genetic diversity is larger in Asia than in Europe, and the pathogen must have gone through a bottleneck, most probably as a result of the species introduction to Europe through a very limited number of individuals.

*Fraxinus mandshurica* has been introduced to Europe consistently during the previous century for amenity plantings. Reports associating symptomatic *F. mandshurica* with *H. fraxineus* in Estonia is rather perplexing since a general lack of symptoms on planted Asian *Fraxinus* species (including *F. mandshurica*) has been widely observed in various parks and natural arboretums in Sweden between the years 2011 and 2014 (M. Cleary, unpublished data) and from a variety of Asian *Fraxinus* species in Denmark^23^, though the possibility cannot be excluded that some symptoms on *F. mandshurica* may be attributable to certain provenances of the species being not well suited to the site^19^, or other predisposing biological factors. The nature of the plasticity of endophytism allows for latent pathogen behaviour to become opportunistic on e.g. physiologically-stressed trees. Purportedly the natural (original) hosts of *H. fraxineus* are *F. mandshurica* and *F. chinensis* since these ash species are the only hosts of *H. fraxineus* thus far reported from Asia^6^,^34^. However, a larger number of *Fraxinus* species are native to Asia and located near or within the natural distribution range of *F. mandshurica* that extends from northeast China to the far east of Russia (see distribution map, Fig. 3). Hence, one cannot exclude the possibility that other Asian *Fraxinus* species also provide a suitable niche for *H. fraxineus*, and which have also been exported to other countries as nursery stock from those regions. In our observations of exotic *Fraxinus* planted in Southern Sweden, *F. mandshurica, F. chinensis, F. floribunda, F. paxiana, F. platypoda*, and *F. sieboldiana* showed no visible crown damage, while European and many North America *Fraxinus* species exhibit varying degrees of characteristic symptoms and crown dieback (M. Cleary, unpublished data). Indeed, more information is needed to clarify the extent of the host range for *H. fraxineus* with other indigineous *Fraxinus* species originating from Asia.

Since *H. fraxineus* was pathogenic on artificial stem-wound inoculations on *F. mandshurica* var. *japonica* seedlings^21^ this might suggest that the active mechanisms that determine susceptibility are found in the leaves following the more ‘natural’ course of establishment via spore germination, appressorium formation and penetration of host tissue^8^. The role of leaf senescence/leaf shed as a mechanism of disease escape in *F. excelsior* trees in Europe is somewhat ambiguous^49^,^50^,^51^. It is obvious that *H. fraxineus* could indeed develop freely in woody tissue^21^, so in theory any inherent traits such as early leaf shed may aid in limiting the establishment of *H. fraxineus* in the stems. Interestingly, senescence in *F. mandshurica* in its native environment occurs earlier and more rapidly compared to several other common broadleaved tree species, with all leaves usually shed by early September^52^, and typically much earlier than *F. excelsior* throughout most of Europe.

The endophytic lifestyle of *H. fraxineus* on *F. mandshurica*, particularly concerning molecular interactions established during penetration, infection and colonization of plant tissue, compared to other hosts such as *F. excelsior* that have not co-evolved with the fungus, can provide clues for better understanding mutualism and pathogenesis and identify commonalities and disparity in host recognition patterns. Inoculation techniques that mimic natural spore infection of *F. excelsior* by *H. fraxineus* using a closed, moist chamber system^8^ are apt for material studies of host-pathogen interactions under conditions more realistic to natural systems (as opposed to artificial wound-inoculations).

Further studies are also needed on the biology of *H. fraxineus* in its native environment on indigineous *Fraxinus* species in Asia to elucidate the time course for sporulation and infection development in leaves and rachises up until leaf senescence and leaf shed. Moreover, the fungal assemblages of eastern *Fraxinus* species and their interaction with *H. fraxineus* warrants further investigation. Of particular interest would be to isolate and test the antagonistic capacity of *Mycosphaeralla* spp. and/or other endophytic fungi uniquely associated to *F. mandshurica* against *H. fraxineus*, and their feasibility as potential biological control agents of *H. fraxineus* on *F. excelsior* in Europe.

## Methods

### Sites and sampling of material

During summer 2012, up to 15 similarly sized asymptomatic leaves (including leaflets and rachises) were randomly collected from the crown of three *F. mandshurica* trees, each growing at three different locations (46°37.148′N, 134°55.545′E; 46°37.319′N, 135°23.0155′E; 44°36.276′N, 134°52.011′E) in mixed forests in the Primorye region, Far East Russia (Fig. 3). Leaves were devoid of any damage symptoms. Leaves were labelled in the field and then dried before storage and processing. In the lab, for each leaf, leaflets and rachises were separated into individual samples, and homogenized in a Lab Wizz micro ball mill (Laarmann, The Netherlands). A total of 75 samples were prepared for sequencing of the fungal community.

### DNA extraction, PCR amplification and sequencing

Total DNA was extracted with CTAB buffer with added 2% (w/v) polyvinylpolypyrrolidone. The ITS region of the rDNA was amplified by PCR using the general primers^53^, gITS7 and ITS4. Each sample was uniquely barcoded. PCR was performed in 50 μl reactions and consisted of the following final concentrations, 0.25 ng μL^−1^ template DNA, 200 μM of dNTPs; 750 μM of MgCl_2_; 0.025 μM polymerase (5 U/μL) (DreamTaq Green, Thermo Scientific, Waltham, USA), and 200 nM of each primer in 1× buffer. Amplifications were performed using the Applied Biosystems 2720 thermal cycler. The PCR program started with denaturation at 95 °C for 5 min, followed by 30 cycles of 95 °C for 30 s, annealing at 56 °C for 30 s and 72 °C for 30 s, followed by a final extension step at 72 °C for 7 min. To check if the PCR was successful, the PCR products were visualized by gel electrophoresis on 1% agarose gel stained with Nancy-520 (Sigma-Aldrich, Sweden). PCR products were purified using Agencourt AMPure XP (Agencourt Bioscience Corp, Massachusetts USA) and with the ‘E.Z.N.A. Cycle-Pure’ kit (Omega) following manufacturer’s instructions. After quantification of PCR products using a Qubit flurometer 2.0 (Life Technologies, Sweden), samples were pooled in an equimolar mix and sent for 454-amplicon sequencing using the GS FLX Titanium chemistry (Macrogen Inc, Seoul, Korea).

### Confirmation of Fraxinus species

A subsample of extracted DNA from the above samples from each of the three sites was selected for determination of *Fraxinus* species. The primers trnH_psbA3/trnHf_05, rbaLa/rbcLa, and matK_390/matK_1326^54^,^55^ were used amplify the nuclear ribosomal intergenic spacer chloroplast regions. Optimal temperature regimes for PCR were established individually by testing amplification success for each primer. Amplifications were performed using the Veriti Thermal Cycler (Applied Biosystems) in 50 μl reactions containing the following final concentrations, 2.5 ng/μl template DNA, 0.025 μM Taq Polymerase (5 U/μL), 200 μM of dNTPs, 750 μM of MgCl_2_, and 0.2 μM of each primer in 1× Buffer. Cycle conditions were initial denaturation at 94 °C for 4 min, followed by 35 cycles of 94 °C for 30 s, annealing for 30 s at 62 °C for trnH_psbA3/trnHf_05, 60 °C for rbaLa/rbcLa, and 50 °C for matK_390/matK_1326, and 72 °C for 30 s, with a final extension at 72 °C for 7 min. PCR products were verified as above and purified using E.Z.N.A. Cycle-Pure kit (Omega) following manufacturer’s instructions. Prepared samples were sequenced by GS FLX Titanium chemistry (Macrogen Inc, Seoul, Korea).

### Sequence analysis

Fungal ITS data derived from 454-amplicon sequencing was processed using the bioinformatics pipeline SCATA available at Department of Forest Mycology and Plant Pathology, Swedish University of Agricultural Sciences (SLU), (http://scata.mykopat.slu.se). Quality filtering removed sequences considered too short (<200 bp), with low mean read quality of <20, and those missing either primers. There were 39,772 sequences that passed the quality control thresholds, and sequences were clustered into operational taxonomic units (OTUs), which we consider to be taxonomically distinct, at 2% dissimilarity. OTUs were identified 1) in the SCATA program by comparing them with reference sequence databases at SLU Department of Forest Mycology and Plant Pathology and UNITE^56^, and 2) by alignment with blastn at GenBank (NCBI). The distinction between rare species and PCR and sequencing errors cannot be easily done and thus, singletons and doubletons were excluded from the dataset. To determine whether there were differences in the community among the three localities, species rarefaction curves were generated from the 20 most frequently occurring OTUs in the vegan package in R. One curve was generated for each site. Nonmetric Multidimensional Scaling (NMDS) ordination was carried out in the vegan package, using the metaMDS function, specifying the Bray-Curtis dissimilarity index, three dimensions and 1000 iterations. The first two dimensions were used to visualize the relative abundances of the 20 most frequently occurring OTUs across the study. Convex hulls were drawn for samples from each site. Shannon-Wiener diversity indices was used to compare the fungal communities of *F. mandshurica* trees among the three localities. Sequences obtained from chloroplast primers were aligned and manually edited using Lasergene software package SeqMan Pro (DNA Star, Madison WI, USA). Species identification was confirmed by comparing the acquired sequences to those deposited in GenBank through blastn search.

### Microsatellite analysis

*Hymenoscyphus fraxineus*-positive samples identified via 454-amplicon sequencing were verified via PCR amplification with specific primers, forward 5′-AGC TGG GGA AAC CTG ACT G-3′, and reverse 5′-ACA CCG CAA GGA CCC TAT C-3′^57^ prior to microsatellite analysis. PCR was performed in 10 μl reaction volumes in a master mix similar to that stated above. The cycling conditions including initial denaturation at 95 °C for 5 min followed by 35 amplification cycles of denaturation at 94 °C for 30 s, annealing at 62 °C for 1 min and extension at 72 °C for 30 s. The reaction was finished by an extension step at 72 °C for 7 min. PCR products were visualized by gel electrophoresis on a 1% agarose gel in SB buffer.

Initially samples were tested using 26 microsatellites (MS) from earlier studies^13^,^14^,^58^ of which 15 amplified fragments in both Asian and European samples were selected for fragment analysis of samples (Table 2). Another set of European *H. fraxineus* samples comprising 15 cultured isolates from Sweden and 10 cultured isolates from Lithuania were used for comparison. All European isolates were derived from diseased tissues of ash (for isolate locations see Supplementary table^15^). PCR amplification was performed in 15 μL reaction volume containing the following final concentrations; 0.025 μM of DreamTaq Polymerase (Thermo Scientific), 1× buffer, 0.2 μM of MgCl_2_, 200 μM of dNTPs, 0.2 or 0.3 μM of each primer (see Table 2) and 1 ng/μl of template DNA. Cycling conditions varied by primer, denaturation for 3 min (5 min for mHp primers) at 95 °C, 35 cycles of 30 s at 95 °C (94 °C for mHp and SSR primers), 30 s at the annealing temperature (see Table 1), 45 s at 72 °C, and a final extension step of 7 min at 72 °C. Successful amplifications were confirmed as previously noted above. Samples were analyzed at SciLife Lab, Uppsala University, Sweden on the ABI 3730XL DNA Analyzer. GeneMarker (Softgenetics) was then used to determine the lengths of the fragments and identify different genotypes at each locus.

### Population genetic analysis

If more than one peak was present in the sample, the dominant peak was recorded. If any sample showed ambiguous result, no record was entered for that particular sample and locus. The results were compiled and multilocus genotypes (MLGs) were created for each sample. If any sample had more than five missing values, the sample was discarded from the analysis. To analyze the allele frequencies, genetic distance and analysis of molecular variance (AMOVA), the Excel-add-in software GenAlEx 6.5 was used. To visualize the genetic differences, a principal coordinate analysis (PCoA) was performed based on the genetic distances using the same software. To infer the common ancestry for the different samples, the Bayesian clustering program Structure version 2.3.4 was used. For each run, the first 500 000 iterations were discarded as burn-in, followed by 500 000 iterations for data collection. The populations were assumed to be in Hardy-Weinberg equilibrium and the alleles were assumed to be independent from each other. The number of populations (K) tested was 1–12 and each was replicated three times. To identify the K value, the average LnP(D) of each K was calculated.

## Additional Information

**How to cite this article**: Cleary, M. *et al.* Friend or foe? Biological and ecological traits of the European ash dieback pathogen *Hymenoscyphus fraxineus* in its native environment. *Sci. Rep.* **6**, 21895; doi: 10.1038/srep21895 (2016).

## Supplementary Material

Supplementary Information

## Figures and Tables

**Figure 1:**
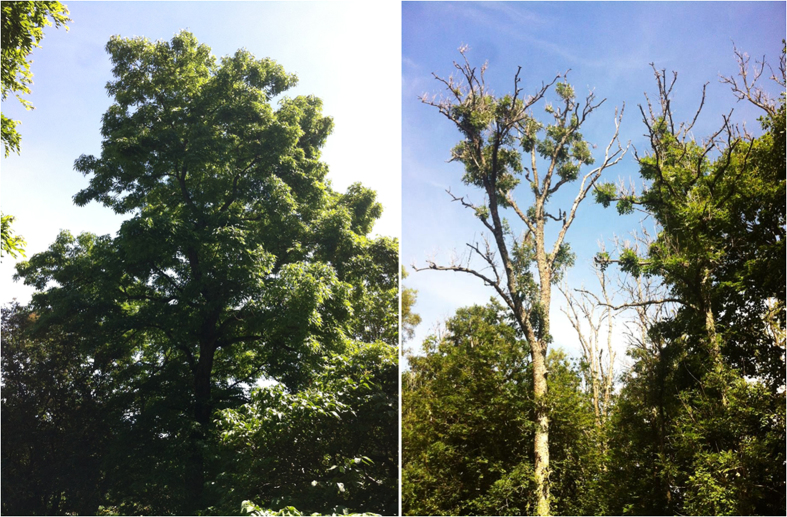
(**a**) *Fraxinus mandshurica* planted in Southern Sweden showing no evidence of crown dieback; (**b**) chronic and progressive dieback symptoms including epicormics branching along the stem of *Fraxinus excelsior* trees infected by *Hymenoscyphus fraxineus* in Southern Sweden. Photos taken by Michelle Cleary.

**Table 1:** Relative abundance, putative identification of fungal taxa, and frequency of detection in leaves of *Fraxinus mandshurica* in Far East Russia, and comparative presence of similar-type taxa to *Fraxinus excelsior* in Europe.

OTU no.	Cluster size	OTU relative abundance	BLAST check	Closest Genbank Accession No.	Max Score	Coverage	% max identity	Frequency of detection on *F. mandshurica* in Far East Russia (%)^a^	Presence on *F. excelsior* in Europe^b^
			Putative taxon						
0	25121	63.16	*Mycosphaerella* sp.	EU167602	403	100	97	99	
1	4620	11.62	*Cladosporium* sp.	KJ921876	444	100	100	84	+
5	1341	3.37	*Phomopsis* sp.	HE774484	466	100	100	80	+
3	1201	3.02	*Phoma* sp.	KJ921933	455	100	100	81	+
2	1062	2.67	*Alternaria alternata*	KF819607	462	100	100	64	+
4	817	2.05	*Dioszegia* sp.	AJ581077	361	100	97	51	
8	759	1.91	*Cryptococcus foliicola*	AY557600	429	100	100	75	+
7	650	1.63	Unknown *Helotiales* sp.	KF636763	435	100	99	47	
6	554	1.39	*Paraphoma* sp.	JX077009	424	100	99	68	
9	342	0.86	*Diaporthe nobilis*	KC343146	459	100	100	40	+
13	254	0.64	*Ramularia* sp.1	GU214690	411	100	98	53	+
15	244	0.61	*Paraconiothyrium* sp.	FN868460	396	100	96	24	
11	187	0.47	*Hymenoscyphus fraxineus*	KJ780099	440	100	100	33	+
18	181	0.46	*Periconia byssoides*	KC954160	455	100	100	33	+
20	173	0.43	*Ramularia* sp.2	GU214692	399	100	97	35	+
14	153	0.38	*Coniozyma leucospermi*	EU552113	440	100	98	45	
12	149	0.37	*Hannaella coprosmaensis*	AF444485	398	100	100	36	
10	146	0.37	Unknown Ascomycota sp.	JN120378	418	100	98	29	
21	90	0.23	*Ramularia* sp.3	JN662315	361	84	99	21	+
17	87	0.22	*Fusarium sp.*	KJ589605	472	100	100	27	+

^a^considering all samples at three locations (n = 75).

^b^fungal communities compared to those earlier reported^9^,^10^,^29^,^30^,^31^,^32^. ‘+’ indicates similar presence of the fungal organism on both *Fraxinus* species in Far East Russia and Europe.

**Table 2:** Primers used in the study, their final primer concentration, annealing temperature and number of alleles shared, fragment sizes found in European and Asian populations respectively.

Primer name^a^	Final primer concentration in PCR^b^	Annealing temperature (°C)	Shared alleles	Only found in European population	Only found in Asian population
mHp_067022	0.2	56	–	250	244, 247, 253
mHp_098984	0.4	56	92	98	89, 95, 105
mHp_108810	0.1	56	–	264, 268, 277	270, 272, 274, 282, 292
mHp_060142	0.15	56	–	164, 170	151, 158, 167, 173
mHp_080495	0.2	56	150	143	146, 148, 152, 156, 160
mHp_080497	0.3	56	–	245, 254	248, 251
mHp_079915	0.25	56	–	184, 201	175, 181, 187, 194, 204, 222
mHp_095481	0.2	56	138	147	134
Chafra_03	0.2	60	189	208	185, 192
Chafra_04	0.2	60	–	101, 111	95, 97, 99, 105 109
Chafra_09	0.2	60	134	144	125, 131, 140
Chafra_13	0.2	60	176, 180	–	166, 170, 172, 178, 184
SSR_211	0.2	64	296	298	292, 294, 314
SSR_38	0.2	64	314	–	–
SSR_58	0.2	57	185	–	–

^a^mHp, Chafra, and SSR primers obtained from previous studies^13^,^14^,^58^.

^b^concentrations of mHp primers were according to that specified^14^.
