# Supplementary material for: Friend or foe? Biological and ecological traits of the European ash dieback pathogen Hymenoscyphus fraxineus in its native environment
Source: Sci Rep. 2016 Feb 22;6:21895. doi: 10.1038/srep21895 (PMC4761999; doi:10.1038/srep21895)
Supplement: Supplementary Information [file srep21895-s1.pdf]

Supplementary Information

Manuscript: “Friend or foe? Biological and ecological traits of the European ash dieback pathogen *Hymenoscyphus fraxineus* in its native environment.”

By : Michelle Cleary, Diem Nguyen, Diana Marčiulynienė, Anna Berlin, Rimvys Vasaitis, and Jan Stenlid

Description of Supplementary Information:
Sequences for fungal OTUs presented in Table 1.

>OTU_0
TTAAGTTCAGCGGGTATCCCTACCTGATCCGAGGTCAACCTTGTGAAAGATTTAACGGCCGCGGCCGCCCGCA
CTCCGAAGCGATTAATGAAATCACAACGCCAGGAGACGGACAGCTCAGCCGGAGACTTTGAGGCGCGCGGGGA
AACCCCGCGGCGCCCAATACCAAGCCAGGCTTGAGTGGTGAAATGACGCTCGAACAGGCATGCCCTCCGGAAT
ACCAGAGGGCGCAATGTGC
>OTU _1
TTAAGTTCAGCGGGTATCCCTACCTGATCCGAGGTCAACCTTAGAAATGGGGTTGTTTTACGGCGTAGCCTCC
CGAACACCCTTTAGCGAATAGTTTCCACAACGCTTAGGGGACAGAAGACCCAGCCGGTCGATTTGAGGCACGC
GGCGGACCGCGTTGCCCAATACCAAGCGAGGCTTGAGTGGTGAAATGACGCTCGAACAGGCATGCCCCCCGGA
ATACCAGGGGGCGCAATGTGC
>OTU_5
TTAAGTTCAGCGGGTATTCCTACCTGATCCGAGGTCAAATTTTCAGAAGTTGGGGGTTTAACGGCAGGGCACC
GCCAGGGCCTTCCAGAGCGAGGGTTTAACTACTGCGCTCGGGGTCCTGGCGAGCTCGCCACTGAATTTCAGGG
CCTGCTTCTTGGGTAAGAAGCAGTGCCCCATCACCAAGCCAGGCTTGAGGGTTGAAATGACGCTCGAACAGGC
ATGCCCTCCGGAATACCAGAGGGCGCAATGTGC
>OTU_3
TTAAGTTCAGCGGGTATCCCTACCTGATCCGAGGTCAAGAGTGTAAAAATGTACTTTTGGACGTCGTCGTTCT
GAGTGCAAAGCGCGAGATGTACTGCGCTCCGAAATCAATACGCCGGCTGCCAATTGTTTTGAGGCGAGTCTAC
GCGCAGAGGCGAGACAAACACCCAACACCAAGCAAGGCTTGAAGGTACAAATGACGCTCGAACAGGCATGCCC
CATGGAATACCAAGGGGCGCAATGTGC
>OTU_2
TTAAGTTCAGCGGGTATCCCTACCTGATCCGAGGTCAAAAGTTGAAAAAAAGGCTTAATGGATGCTAGACCTT
TGCTGATAGAGAGTGCGACTTGTGCTGCGCTCCGAAACCAGTAGGCCGGCTGCCAATTACTTTAAGGCGAGTC
TCCAGCAAAGCTAGAGACAAGACGCCCAACACCAAGCAAAGCTTGAGGGTACAAATGACGCTCGAACAGGCAT
GCCCTTTGGAATACCAAAGGGCGCAATGTGC
>OTU_4
TTAAGTTCAGCGGGTAGTCCTACCTGATTTGAGGTCAGAATATAAAGTGTAGTTAGAGTGGAGGGTTGCGAGA
ACTTATTACGCATTCCACTTAGACATTTAAGGCGAGCCAGTACTGGCAAACACCCAAAGCACAACTATAGAAA
ACTATAGAGGTGAGGTTTATAAGACACTCAAACAGGCATGCCCTTCGGAATACCAAAGGGCGCAATCTGC
>OTU_8
TTAAGTTCAGCGGGTAGCCCTACCTGATTTGAGGCCAGATCATGAGATATGTGGGGTTATCAGCCACTTAGAA
GGATGAAACGTATTACATCCAAAGTTGCTTATGCTTTTAAGGTGAGCCAGTGACGGCAAACACCCAATCCACC
ACCGCTCGGGCAAAAACCCAAGTGGGGTGAGGTTTCATGACACTCAAACAGGCATGCCCTTCGGAATACCAAA
GGGCGCAAGGTGC
>OTU_7
TTAAGTTCAGCGGGTATCCCTACCTGATCCGAGGTCAACCTGTAAAAATTGGGGGTTGCTGGCAAGTAAACCT
ACCGGACTCAAACGCGAGGAGTATTACTACGCGTAGAGCCGACAGGCACCGCCACTGATTTAGAGGCCGCGG
AACCGCGAACCCCAAGACCAAGCGTGAGCTTGAGTGGTTGTAATGACGCTCGAACAGGCATGCCCCCCGGAAT
ACCAGAGGGCGCAATGTGC
>OTU_6
TTAAGTTCAGCGGGTATCCCTACCTGATCCGAGGTCAAAAGTGAAAAAGGCTTGTGGAAGCGGGCGTGTAGCC
TTGAATCGCGACTTGTGCTGCGCTTCAAAACCAAACACTGGCTGCCAATTACTTTAAGGCGAGTCCCTCGGGA
CAAACACCCAACACCAAGCAAAGCTTGAAGGTACAAATGACGCTCGAACAGGCATGCCCCATGGAATACCAAG
GGGCGCAATGTGC
>OTU_9
TTAAGTTCAGCGGGTATTCCTACCTGATCCGAGGTCAAATTTTCAGAAGTTGGGGGTTTAACGGCAGGGCACC
GCCAGGGCCTTCCAAAGCGAGGGTTTAACTACTGCGCTCGGGGTCCTGGCGAGCTCGCCACTGAATTTCAGGG
CCTGCCCTTTTACAGGCAGTGCCCCATCACCAAGCCAGGCTTGAGGGTTGAAATGACGCTCGAACAGGCATGC
CCTCCGGAATACCAGAGGGCGCAATGTGC
>OTU_13
TTAAGTTCAGCGGGTATCCCTACCTGATCCGAGGTCAACCTTTGAAAGATTTAACGGCCGCGACGACTCGAAC
TCCACTGCGAATTGAATTGCCACAACGCTGGGAGACGAACCGCTCAGCCGGAGACTTTGAGGCGCGCGAGGGA
CTCGCGACGCCCAATACCAAGCGAGGCTTGAGTGGTGAAATGACGCTCGAACAGGCATGCCCCCCGGAATACC
AGGGGGCGCAATGTGC

>OTU_15
TTAAGTTCAGCGGGTATCCCTACCTGATCCGAGGTCAAAGACGGTAAATTGCTTGCTGGACGCAGGCCGCAGC
ACCTCAAGAAGCGCAATGTGCTGCGCGAGAGGTGGCGGAGACCGCTGCCAATGAATTTGGGGCGAGTCCACGC
GCAAAGGCGGGACAGACGCCCAACACCAAGCAGAGCTTGAGGGTGTAGATGACGCTCGAACAGGCATGCCCCA
TGGAATACCAAGGGGCGCAATGTGC
>OTU_11
TTAAGTTCAGCGGGTATCCCTACCTGATCCGAGGTCAACCCTAGAGAGTTGGGGGTTGCTGGCAAGACACCGC
AAGGACCCTATCGCGAGAAGAATTACTACGCGTAGAGCCTTAGGGCACCGCCACTGGTTTTAAGGCCCGCCCA
AAGGCGCAGCCCAAGACCCCGCCGGAGCGGGAGTTGGTCTAAATGACGCTCGAACAGGCATGCCCCCCGGAAT
ACCAAGGGGCGCAATGTGC
>OTU_18
TTAAGTTCAGCGGGTATCCCTACCTGATCCGAGGTCAAGTCATGAAATTGCTTGCTGGACGCTGATCCGCCGG
GGCTCCAAGAAGCGCAAATGTGCTGCGCGAGAAGCCGACGAGACCGCTGCCAATGACTTTGAGGCGAGTCCGC
GCACGGAGGCGGGACAGACGCCCAACACCAAGCAGAGCTTGAGGGTGTAAATGACGCTCGAACAGGCATGCCC
CACGGAATACCGAGGGGCGCAATGTGC
>OTU_20
TTAAGTTCAGCGGGTATCCCTACCTGATCCGAGGTCAACCTTTGAAAGATTTAACGGCCGCGACGTCTCACAC
TCCACTGCGAAATAGTTGCCACAACGCTGGGAGATGAACCGTACAGCCGGAGACTTTGAGGCGCGCGAGAGAC
TCGCGACGCCCAATACCAAGCGAGGCTTGAGTGGTGAAATGACGCTCGAACAGGCATGCCCCCCGGAATACCA
GGGGGCGCAATGTGC
>OTU_14
TTAAGTTCAGCGGGTATCCCTACCTGATCCGAGGTCAACCTGAAAAAATAAAAGGTTTTACGGCAATGAGATC
TCAACAAGCTTATAAGACGTTTTGATTTAACTCTACTACGCCCGAAGTTGGAGAAACCCCGCCGAGGTCTTCG
AGGCACGCCCGTCTTTCGACGGACGATGCCCAATACCAAGCAGTGCTTGAGTGGTGTAATGACGCTCGAACAG
GCATGCCCCTCGGAATACCAAGGGGCGCAATGTGC
>OTU_12
TTAAGTTCAGCGGGTAGTCCTACCTGATTTGAGGTCAGAGATAAAAGTGTATTAGAGTGGAGGGAGCGAAAAC
TTATTACGCATTCCACTTAGACATTTAAGACGAGCCAAAGCATGGCAGACGTCCAAAGCACCCAACTCGGAAA
CCCGAGAGGATGAGTCTATATGACACTCAAACAGGCATGCTCTTCGGAATACCAAAGAGCGCAATGTGC
>OTU_10
TTAAGTTCAGCGGGTATCCCTACCTGATCCGAGGTCAACCTTTAAAAAATTGGGGGTTACTGGCAAGACACCA
CCGAGACCCTATAGCGAGAAGAATTACTACGCTCAGAGCTCGAGGGCACCGCCACTGGGTTTAGAGGCTGCGG
AATCGCAGGCCCCAAGACCAAGCCGAAGCTTGATGGGTTGAAATGACGCTCGAACAGGCATGCCCCGCGGAAT
ACCACGGGGCGCAATGTGC
>OTU_21
TTAAGTTCAGCGGGTATCCCTACCTGATCCGAGGTCAACCTGAAAAAAGAGGTTGGACGGCACACTCCGACCG
GCCACCGACGCGAGACAGATGCCACAACGCGAGGGGACGGACGGCGTGGCCGGCGACATTGGGGCGGGCGGCC
AGAAGCCGCGGCGCCCAACACCCAGCGGGGCTGGATTGGTGACAATGACGCTCGAACAGGCATGCCCCCCGGA
ATACCAGAGGGCGCAATGTGC
>OTU_17
TTAAGTTCAGCGGGTATTCCTACCTGATCCGAGGTCAACATTCAGAAGTTGGGGTTTTACGGCATGGCCGCGC
CGCGTTCCAGTTGCGAGGTGTTAGCTACTACGCAATGGAGGCTGCAGCGAGACCGCCAATGTATTTCGGGGGC
GGCACCGCCAGAAGGCAGAGCCGATCCCCAACACCAAACCCGGGGGCTTGAGGGTTGAAATGACGCTCGAACA
GGCATGCCCGCCGGAATACCAGCGGGCGCAATGTGC
